# Effect of ovariectomy on proximal tibia metaphysis and lumbar vertebral body in common marmoset monkeys

**DOI:** 10.5194/pb-6-65-2019

**Published:** 2019-07-12

**Authors:** Christina Schlumbohm, Dana Seidlová-Wuttke, Eberhard Fuchs

**Affiliations:** 1 German Primate Center, Leibniz Institute for Primate Research, 37077 Göttingen, Germany; 2 Department of Endocrinology, University Medical Center Göttingen, 37075 Göttingen, Germany

## Abstract

This study aimed to investigate the effect of estrogen withdrawal on bone tissue in adult female marmoset monkeys. In a 1-year follow-up study we used quantitative computer tomography to measure total bone mineral density (BMD) of the proximal tibia and the second-last lumbar vertebral body (L5/L6) before and 1, 3, 6, and 12 months after ovariectomy. Body mass did not significantly change during the 1-year observation period. However, a significant decline of total BMD after ovariectomy was observed in the proximal tibia but not in L5/L6. In addition, regression analysis showed a significant positive relationship between BMD and body mass in both tibia and L5/L6. The results of our study support the idea that ovariectomized marmoset monkeys may serve as a model to investigate bone loss related to decline of estrogen production.

## 1 Introduction

More than 70 years ago the link between bone loss and estrogen depletion in women was first described (Albright et al., 1940; Reifenstein and Albright, 1947). Today we know that in about 20 % of women, osteoporosis develops after menopause when estrogen production ceases because of high bone turnover and excess resorption (IOF, 2018). In preclinical osteoporosis research, the ovariectomized rat is an accepted and valuable animal model (Levolas et al., 2008; Giardino et al., 1993; Kalu, 1991). However, in the *Guideline on the evaluation of new medical products in the treatment of primary osteoporosis* published by the European Medicines Agency (EMEA; see http://www.emea.europa.eu/pdfs/human/ewp/055295en.pdf, last access: 24 June 2019) it is stated that new substances for treatment of postmenopausal osteoporosis should be tested in at least two species, one of which should be the ovariectomized rat and the other a mammal with evaluable cortical bone remodeling. Nonhuman primates, sheep and pigs, are suggested as a second animal model by the EMEA.

Among nonhuman primates, the common marmoset monkey (*Callithrix jacchus*), a small-bodied non-endangered New World primate, has been shown to be a valuable model for studying aging, reproduction, neuroscience, toxicology, and infectious diseases (Mansfield, 2003; Tardif et al., 2008; Carrion Jr. and Patterson, 2012; 't Hart et al., 2012). In a recent paper it was suggested that orchidectomized male common marmosets are a suitable model to study the development of bone mineral loss related to androgen deficiency (Seidlová-Wuttke et al., 2008). This result is supported by the findings that both osteonal remodeling and bone metabolism are similar to that of humans (Angeliewa et al., 2004; Bagi et al., 2007; Grohmann et al., 2012) and the antiosteoporosis drugs ibandronate (Angeliewa et al., 2004) and alendronate (Bagi et al., 2007) increase bone mass in intact marmosets.

The question of whether induction of estrogen deficiency would induce loss of bone mineral density in adult female marmoset monkeys was addressed in a recent study by Saltzman et al. (2018). The reproductive physiology in adult female marmosets is significantly triggered by social status. Physiologically, subordinate individuals are characterized by prolonged periods of anovulation which are, among others, associated with very low concentrations of estrogen and their reproductive activity may remain suppressed for 2 years or more (Abbott and George, 1991). In their present study Saltzman et al. (2018) show that socially or surgically induced hypoestrogenism is not associated with adverse skeletal consequences such as lower bone mineral density in the lumbar vertebrae (L5/L6) of female marmosets. This is a very interesting result as it demonstrates for the first time in female primates the conservation of bone mass despite estrogen deficiency. In line with these findings we could not observe in our present 1-year follow-up study a significant decline of total bone mineral density after ovariectomy (ovx) in L5/L6. However, a significant decline of total bone mineral density after ovariectomy was observed in the proximal tibia raising the question of which parts of the skeleton may be suitable or even better to study the effects of estrogen deficiency in female marmosets.

## 2 Materials and methods

All experiments were performed in accordance with the European Communities Council Directive 86/609/EEC and the German legislation on animal rights and welfare and were approved by the Lower Saxony Federal State Office for Consumer Protection and Food Safety, Oldenburg, Germany. Whenever applicable, ARRIVE (Animal Research: Reporting of In Vivo Experiments) guidelines were followed.

### 2.1 Experimental animals

Six adult female common marmoset monkeys (*Callithrix jacchus*) participating in this study were born and raised at the German Primate Center, Göttingen, Germany, and served as active breeders until ovariectomy. From last parturition (at least 6 months before the study) until ovariectomy, pregnancies were prevented by intramuscular injections of a PGF2α analogue (2.5 µg cloprostenol per animal; Estrumate^®^, Essex-Tierarznei GmbH, Munich, Germany) every 24 d. The animals were 3–7 years of age when the experiment started (mean±SD, 4.8±1.4 years). The marmoset monkeys were housed in opposite-sex pairs in stainless-steel wire-mesh cages including wooden platforms, perches, hammocks, and a wooden nest box to stimulate and promote species-typical behavior such as scent-marking, climbing, and foraging. The floor under the cages was covered with paper sheets. Urine, feces, and leftover feed were removed daily by replacing the paper sheets.

The experimental rooms and the cages were cleaned at weekly intervals and disinfected using water and Biguacid (Antiseptica, Brauweiler (Pulheim), Germany). The room temperature was maintained at 26±1.5 ${}^{\circ }C$, and the relative humidity was kept between 60 % and 80 %. These parameters were under daily control. Artificial light was set to provide a cycle of 12 h light and 12 h dark, with lights on at 07:30 CET. The air in the room was changed about 8 times per hour and filtered adequately. All materials were changed regularly, cleaned, and sterilized.

A pelleted marmoset diet (ssniff Spezialdiäten GmbH, Soest, Germany; http://www.ssniff.de, last access: 24 June 2019) was fed ad libitum. This diet (sniff^®^ V3843-000) contained (per kilogram of dry matter) 26 % crude protein, 7 % fat, 2.5 % crude fiber, 43.8 % starch and saccharides, 1 % calcium (Ca), 0.7 % inorganic phosphate ($P_{i}$), 3000 IU (international units) vitamin D3, and 15.6 MJ of digestible energy. In addition, a mash which contained in the dry matter 21 % crude protein, 14 % fat, 10 % crude fiber, 41 % starch and saccharides, 0.95 % Ca, 0.67 % $P_{i}$, and (per kilogram) 5500 IU vitamin D3 and 17.9 MJ digestible energy was served in the morning. Each animal was offered 20 g of the mash with a moisture content of 52 %. In the afternoon, each animal received 30 g of clean-cut fruits or vegetables.

Water was offered ad libitum via a drinking bottle.

To keep the number of experimental animals at a minimum we decided not to use a control group. Instead, the animals served as their own controls and returned to the animal colony after the observation time of 12 months.

To evaluate the influence of age and nonbreeding on body mass data, 23 intact females (age 2–8 years) from the same colony housed in opposite-sex pairs and treated with PGF2α analogue at 24 d intervals were evaluated.

### 2.2 Ovariectomy

Bilateral ovariectomy was performed under general anesthesia using a ventral midline approach and approved standard methods. The success of the ovariectomy was verified by measurement of serum 17β-estradiol concentrations. All animals had 17β-estradiol concentrations <20.0 $\mathrm{pg}\;{\mathrm{mL}}^{-1}$ after ovariectomy (data not shown). Body mass was determined with a bench scale (FCB, Kern, Balingen–Frommern, Germany) and blood samples were collected after the morning feed between 09:00 CET and noon on the day before the quantitative computerized tomography measurement. Blood samples were collected from the femoral vein via a syringe, immediately transferred into a tube (S-Monovette^®^ Serum-Gel, Sarstedt, Nümbrecht, Germany), centrifuged, and the serum was stored frozen until analysis. The 17β-estradiol concentration was measured by radioimmunoassay (RIA; DSL-43100; Diagnostic Systems Laboratories Inc., Webster, TX, USA).

### 2.3 Quantitative computerized tomography

Quantitative computerized tomography (qCT) was performed 1–2 weeks before and 1, 3, 6, and 12 months after ovariectomy. Tomography was performed in the morning under general anesthesia after an overnight food, but not water, withdrawal. For anesthesia, animals received intramuscular injections of 10 $\mathrm{mg}\;{\mathrm{kg}}^{-1}$ alphaxalone, but not more than 5 mg per animal (Alfaxan^®^; Jurox Limited, Crawley, UK; 10 $\mathrm{mg}\;{\mathrm{mL}}^{-1}$), glycopyrronium bromide (0.01 mg per animal, Robinul^®^; Riemser, Greifswald, Germany; 0.2 $\mathrm{mg}\;{\mathrm{mL}}^{-1}$), and diazepam (0.125 mg per animal, Diazepam-Ratiopharm^®^, Ulm, Germany; 10 mg/2 mL injectable solution). The qCT measurements of the proximal tibia and lumbar vertebrae were performed successively and lasted 12 min each.

**Figure 1:**
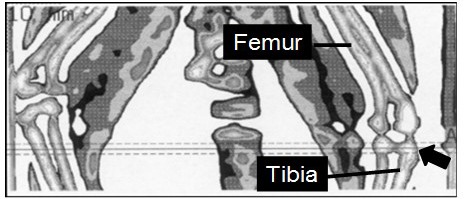
Tomographic scout view of the distal femur and the proximal tibia of a marmoset monkey recorded by quantitative computer tomography. The arrow points to the left proximal tibia. Radiographs were taken in the reference line (solid line) and 1.0 mm proximally and distally from it (dashed lines). The image refers to a square of 2.8cm×6 cm.

qCT was performed using the XCT 2000 (Type 803100, Stratec Inc., Pforzheim, Germany). For the first scan of the proximal metaphysis of the left tibia, the scanner was positioned at the knee bend and a coronal computed radiograph (scout view) in the distal direction was generated. The scout view was used to position the scanner at the site of measurement, as illustrated in Fig. 1. Three tomographic slices, one in the reference line, one 1.0 mm proximal, and one 1.0 mm distal to it, were recorded.

**Figure 2:**
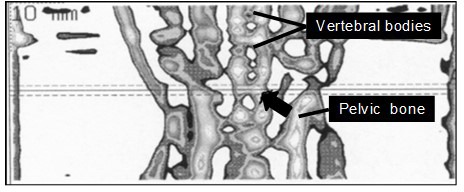
Tomographic scout view of the lower part of the body of a marmoset monkey showing bone tissue of the pelvic, lumbar, and sacral spine. The arrow points to the position of the reference line (solid line) which equals the center of the second-last lumbar vertebral body. Rostral and caudal of this line, additional cross sections were taken at a distance of 1.0 mm from the reference line (dashed lines). The image refers to a square of 2.8×cm6 cm.

Image acquisition, processing, and calculation of the results were performed using the software package XCT 5.40 (Stratec Inc.). The software separates at the outer borderline of the bone all voxels located in the soft tissue below a defined density threshold (280 $\mathrm{mg}\;{\mathrm{cm}}^{-3}$). Because of the strong contrast in X-ray attenuation between the outer cortical shell of bone and the surrounding soft tissue, the region of interest (ROI) gives reliable data on the total area and total mineral density of bone in a defined region. Density and area were measured in the second-last lumbar vertebra and in the proximal tibia in three consecutive cross sections at 1 mm distance apart (Figs. 1 and 2).

Because the number of lumbar vertebrae in marmoset monkeys is 6 or 7 (Wagner and Kirberger, 2005; Casteleyn et al., 2012), and the individual number of lumbar vertebrae was not known, we could not specify whether L5 or L6 was scanned. The reference line of the scanner was positioned in the center of the second-last vertebral body with cross sections taken 1.0 mm proximal and caudal of the center (Fig. 2). The reproducibility of qCT was tested by consecutive measurements of the same bone location before and after repositioning of the animal within the scanner. Reproducibility of two measurements was always better than 95 %.

Because appositional bone growth is absent in adult marmoset monkeys (Bagi et al., 2007), the perimeter of a given cross section is expected to remain relatively constant over time. Thus, bone perimeter is not expected to differ between measurements when the scanner positioning is the same. It was postulated that reliable data could be expected when the mean bone circumference showed no tendency to decrease or increase significantly (p>0.1) between measurements at different times.

### 2.4 Data analysis and statistics

Linear regression analysis and paired t test were used to analyze the data (SigmaStat 3.0, SyStat Software GmbH, Erkrath, Germany).

## 3 Results

### 3.1 Body mass

**Figure 3:**
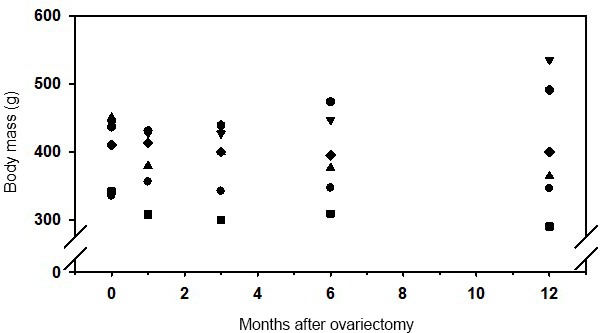
Body mass of six female marmoset monkeys before and during 12 months after ovariectomy.

Body mass was not significantly affected during the 1-year observation period (Fig. 3). However, 1 month after ovariectomy a small, but not significant, decrease in body mass was observed. Obviously the observed variation of body mass increased with time and was highest 12 months after ovx.

**Figure A1:**
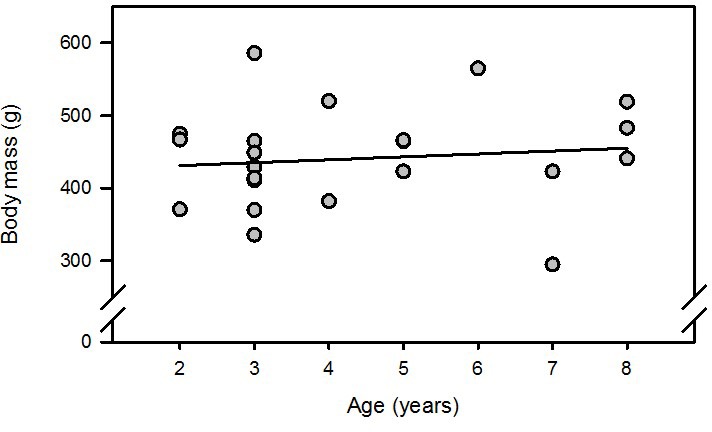
Regression analysis of body mass versus age of 23 intact PGF2α-analogue-treated female marmoset monkeys 6 months from their last parturition. R=0.12; n.s. (not significant).

**Figure A2:**
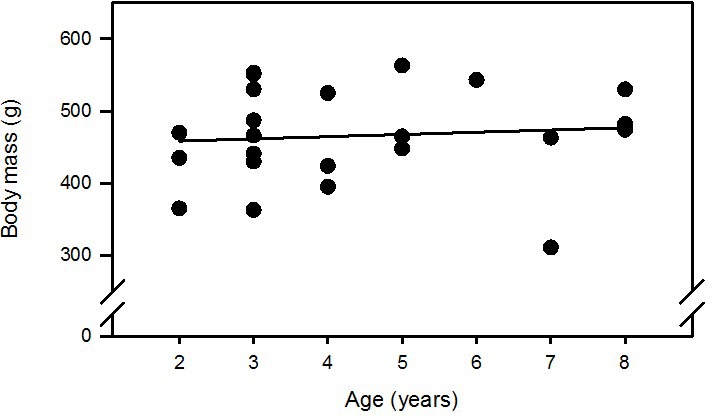
Regression analysis of body mass versus age of 23 intact PGF2α-analogue-treated female marmoset monkeys 18 months from their last parturition. R=0.09; n.s. (not significant).

**Figure A3:**
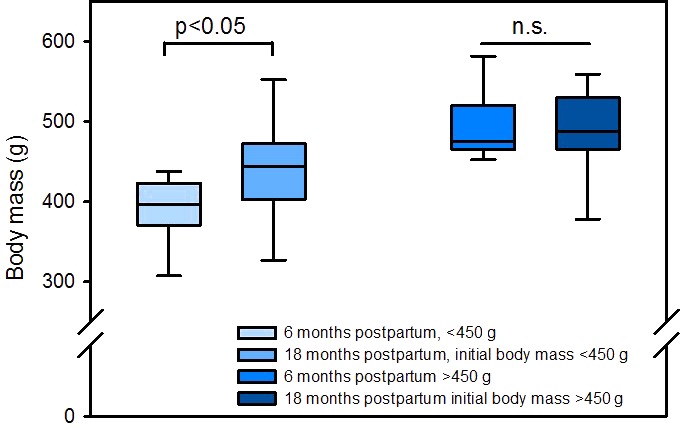
Body mass in 23 intact PGF2α-analogue-treated female marmoset monkeys 6 and 18 months from the last parturition. Data from animals with initial body mass <450 g (n=12) or >450 g (n=11) are shown separately (Median, 5 %, 25 %, 75 %, 95 % percentiles).

Analysis of body mass development in 23 intact females from the same colony housed in opposite-sex pairs and treated with PGF2α analogue revealed that (i) age did not affect body mass (Figs. A1 and A2 in the Appendix) and (ii) variability of body mass increased with time in these nonbreeders (Fig. A3).

### 3.2 Effect of ovariectomy on the proximal tibia

As mentioned above, the bone perimeter was used as a measure of reproducibility of the scanning position. The perimeters of the proximal tibia at the scanning positions did not differ between months 0, 1, 3, 6, and 12 after ovariectomy (data not shown).

**Figure 4:**
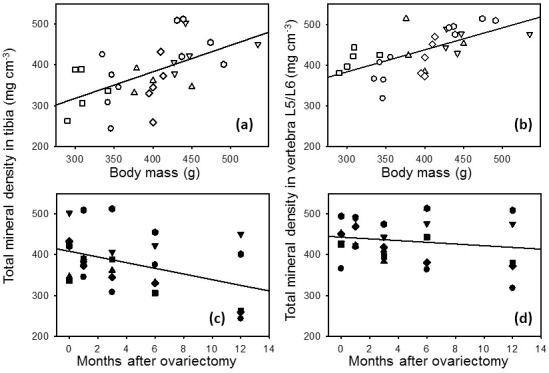
Total mineral density and regression line in proximal tibia vs. body mass **(a)** or vs. months after ovariectomy **(b)** and total mineral density in the second-last lumbar vertebrae (L5/L6) and regression line vs. body mass **(c)** or vs. months after ovariectomy **(d)** in six female marmoset monkeys. Corresponding statistical data are summarized in Table 1.

**Figure 5:**
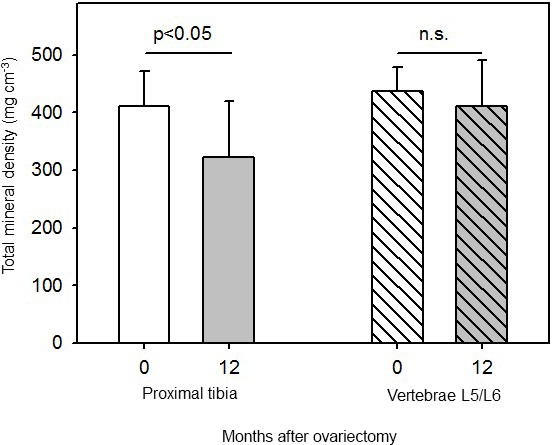
Total mineral density in proximal tibia and in the second-last lumbar vertebrae (L5/L6) in six female marmoset monkeys before (0 months) and 12 months after ovariectomy (mean±SD, paired t test).

Total bone mineral density (BMD) of the tibia declined linearly with time and reached its lowest value at 12 months after ovx (Fig. 4b). Total tibial BMD was significantly (p<0.05) lower at 12 months after ovx than before ovx (Fig. 5). Total BMD in tibia declined in all animals. Perimeters of the bone areas used for total BMD measurements did not differ (20.7±2.2 and 20.4±1.4 mm before and 12 months after ovx, respectively). Loss of BMD was not related to initial body mass (data not shown).

### 3.3 Effects of ovariectomy on the lumbar vertebrae

There was no significant effect on total BMD in L5/L6 before (0 months) and 12 months after ovariectomy (Figs. 4d, 5). In addition, perimeters of the cross sections did not differ before (0 months) and 12 months after ovx (22.1±1.7 and 22.2±1.6 mm, respectively).

### 3.4 Effect of body mass on proximal tibia and lumbar vertebrae

Total BMD in the proximal tibia and in the lumbar vertebrae increased with increasing body mass (Fig. 4a and c). Both relations, i.e., between total BMD in proximal tibia and body mass as well as total BMD in the lumbar vertebrae and body mass, could be described by linear regression equations. For these linear regressions all data of total BMD in tibia or L5/L6 were related to the respective body mass data. In these one factorial approaches the time after ovx was not taken into account. By minimizing the squares of deviations, the best fitting regression line was calculated. For total BMD vs. body mass this equation, for example, had the following form: total BMD${}_{\mathrm{tibia}}\;(\mathrm{mg}\;{\mathrm{cm}}^{-3})=122.023+(0.653\cdot \text{body mass}$). Statistics of this type of equation are summarized in Table 1.

**Table 1:** Regression statistics of total mineral density in proximal tibia and in the second-last lumbar vertebrae (L5/L6) vs. body mass or vs. months after ovariectomy. Data plots and regression lines are shown in Fig. 4a–d.

	Total mineral density tibia				Total mineral density L5/L6			
	Panel in Fig. 4	R	Power	p value	Panel in Fig. 4	R	Power	p value
Body mass	(a)	0.56	0.90	0.002	(c)	0.63	0.97	0.001
Months after ovariectomy	(b)	0.42	0.62	0.001	(d)	0.17	0.14	0.001

## 4 Discussion

The two main results of this investigation are that (1) female marmoset monkeys lose bone mass in the proximal tibia within 1 year after ovariectomy (Figs. 4b and 5) but not in the lumbar vertebra (Figs. 4d and 5) and that (2) body mass is a strong predictor of bone mass in the lumbar vertebrae and in the tibia.

Body mass was unaffected by ovx (Fig. 3). This is in line with a recent report showing that body mass did not change 6 to 7 months following ovariectomy (Saltzman et al., 2018). Thus, the changes in total bone mineral density observed in this study after ovx were not secondary to changes in body mass.

Our result that the lumbar vertebrae did not respond to ovariectomy within 12 months after ovx is consistent with the findings of Colman and coworkers, who reported that, even after 2 years, ovariectomy did not affect vertebral bone mineral density in marmoset monkeys (Colman et al., 1997). A similar finding was reported recently by Saltzman et al. (2018).

In women, high body mass protects to some extent from postmenopausal bone loss (Rico et al., 2002; Cifuentes et al., 2003). Because women produce increasing amounts of estrogen with increasing size of the fat depots (Baglietto et al., 2009) their body mass had a greater impact on BMD than the interval since menopause (Cifuentes et al., 2003). One-way linear regression analysis (Fig. 4a, c, Table 1) showed that a high body mass had a positive significant effect on BMD in marmoset monkeys also.

From this we conclude that lean marmoset monkeys may be more suitable for studying bone loss related to estrogen deficiency than overweight or obese animals and that weight gain during such studies should be avoided in this setting. Our current data show that estrogen withdrawal in female marmoset monkeys induces loss of bone mass in the axial skeleton as it is generally observed in postmenopausal women (Ji and Yu, 2015). The lack of loss of bone mass in the vertebral bodies differs from what is seen in postmenopausal women.

In contrast to reports in rats (Bonnet et al., 2006), we found that in adult female marmoset monkeys BMD does not correlate with age (data not shown) and that bone perimeter remained constant during the experimental period. Rat models show an increase in circumference (Kalu, 1984) and length (Waarsing et al., 2006) of the tibia with age due to appositional bone growth. This is not the case in the marmoset tibia. This view is supported by the finding that epiphyseal closure in the long bones occurs not later than the age of 1.8 years in marmoset monkeys (Kohn et al., 1997). The absence of a significant relationship between bone perimeter and time is one more similarity to human bone biology (Grohmann et al., 2012). Several studies have been carried out in ovariectomized or postmenopausal nonhuman Old World monkeys such as baboons and macaques (Brommage, 2001; Turner, 2001). These species lose bone mass after castration (Jerome et al., 1995; Hordon et al., 2006). However, osteoporosis studies in Old World monkeys are long lasting and may be confounded by age-related effects (Lundon et al., 1994; Cahoon et al., 1996; Champ et al., 1996). For example, in rhesus monkeys, closure of the epiphyseal plate takes place at about 6 years of age but further bone mass is gained up to 9–15 years of age and is lost thereafter (Jerome et al., 1995; Cahoon et al., 1996; Champ et al., 1996). Compared with macaques, marmoset monkeys have an earlier puberty and sexual maturity (Abbott et al., 2003) and presumably achieve earlier peak bone mass. In addition, the animals are easy to breed and to handle under controlled laboratory housing conditions. Therefore, they may have advantages over macaques in preclinical osteoporosis research.

In a recent study, Saltzman and coworkers reported that estrogen depletion is not associated with lower bone mass in female marmoset monkeys (Saltzman et al., 2018). However, their conclusion was drawn from studying lumbar vertebrae L5/L6. Our results support the view that the effect of estrogen withdrawal is not necessarily the same in all localizations of the skeleton. In line with their findings we also could not observe an effect of ovariectomy on total bone mineral density of L5/L6 (Fig. 5). Our study, however, shows that bone tissue of the tibia of adult female marmoset monkeys is sensitive to estrogen withdrawal and is setting up the question of whether other parts of the skeleton may also be suitable or even better to study the effects of ovariectomy.

The crucial differences between our study and that by Saltzman and coworkers are the use of two different imaging techniques: in this case qCT versus DXA (dual X-ray absorptiometry), a 12-month versus a 6–7-month duration of surgically induced estrogen depletion, and housing the animals in opposite-sex pairs versus group housing with sexually dominant and subordinate females. Depending on the imaging technique and measuring site, different information on bone quality is obtained. Amstrup et al. (2016) investigated in postmenopausal women to what extend the results from these imaging techniques correlate. They found a good correlation between the methods when assessing the same skeletal site. However, when assessing correlations between different sites, central and distal sites, the associations were only weak to moderate. This may explain why the results of our study and that by Saltzman and coworkers reveal similar results for the effect of long-term estrogen depletion on bone mineral density in lumbar vertebrae L5/L6. It would be interesting to see if a similar agreement – in this case reduction of bone mineral density – can be found when measuring the proximal tibia with DXA.

The results presented in this 1-year follow-up study support the idea that the ovariectomized marmoset monkey is a promising nonrodent model for preclinical testing of anti-osteoporosis drugs. However, for the development of a standardized preclinical model we will need more information about the marmoset skeleton, which should be studied in more detail with respect to localizations of post-castrational bone loss; extent of pre- and post-castrational remodeling; and distributions of cytokine, steroid, and peptide hormone receptors. Undoubtedly, bone of marmoset monkeys shows close similarities to human bone (Bagi et al., 2007; Grohmann et al., 2012), responds to standard therapeutics (Bagi et al., 2007), and is a valuable addition for preclinical research. Moreover, other menopausal symptoms such as hot flashes and sleep disturbances could also show to be inducible by estrogen withdrawal in the marmoset monkey (Gervais et al., 2016). These findings open an additional window for holistic approaches in preclinical drug testing which in the best case can answer more than one question about the efficacy of new therapies.

## Supplement

10.5194/pb-6-65-2019-supplement The supplement related to this article is available online at: https://doi.org/10.5194/pb-6-65-2019-supplement.

## Data Availability

Data used in this paper are available in the Supplement.
