# Supplementary material for: Effect of ovariectomy on proximal tibia metaphysis and lumbar vertebral body in common marmoset monkeys
Source: Primate Biol. 2019 Jul 12;6(2):65–73. doi: 10.5194/pb-6-65-2019 (PMC7041521; doi:10.5194/pb-6-65-2019)
Supplement: The supplement related to this article is available online at: https://doi.org/10.5194/pb-6-65-2019-supplement. [file pb-6-65-supplement.zip › pb-6-65-2019-supplement-title-page.pdf]

Supplement of Primate Biol., 6, 65–73, 2019
https://doi.org/10.5194/pb-6-65-2019-supplement
© Author(s) 2019. This work is distributed under
the Creative Commons Attribution 4.0 License.

Supplement of

Effect of ovariectomy on proximal tibia metaphysis and lumbar vertebral body in common marmoset monkeys

Christina Schlumbohm et al.

Correspondence to: Christina Schlumbohm (cschlumb@gmx.de)

- pb-6-65-2019-supplement-title-page.pdf
- Data for Primate Biology_Figures_3to5.xls
- data_additional_Figures 1-3.xls

The copyright of individual parts of the supplement might differ from the CC BY 4.0 License.
